# *Hippocampus* and *cornu ammonis*: mythonyms that prevail in *Terminologia Anatomica*, *Terminologia Neuroanatomica*, and *Terminologia Histologica*

**DOI:** 10.3389/fnana.2025.1582837

**Published:** 2025-04-03

**Authors:** Jhonatan Duque-Colorado, Laura García-Orozco, Alicia Castillo-Martínez, Mariano del Sol

**Affiliations:** ^1^Doctoral Program in Morphological Sciences, Faculty of Medicine, Universidad de La Frontera, Temuco, Chile; ^2^Faculty of Medicine, Center of Excellence in Morphological and Surgical Studies (CEMyQ), Universidad de La Frontera, Temuco, Chile; ^3^Faculty of Medicine, Universidad Nacional Autónoma de México, México City, Mexico

**Keywords:** neuroanatomy, brain, cerebrum, hippocampus, cornu ammonis

## Abstract

Julius Caesar Arantius first described the hippocampus and proposed the term *hippocampum*. Years later, French anatomists called the structure ram’s horns, and a decade later, it was named cornu ammonis. Although both concepts were first associated with the same structure, their use has expanded to include different but related structures. This situation can make understanding and applying the terminology more difficult. The objective of this work was to determine the presence of the terms *hippocampus*, *cornu ammonis* and their variants in *Terminologia Anatomica*, *Terminologia Neuroanatomica*, and *Terminologia Histologica*, evaluating their congruence in said terminologies, in addition to examining the etymology of both terms. We searched *Terminologia Anatomica*, *Terminologia Neuroanatomica*, and *Terminologia Histologica* for terms containing the concepts *hippocampus*, *cornu ammonis*, and their derivatives. We analyzed the terms *hippocampus* and *cornu ammonis* from their etymology by examining several Latin texts. This analysis included the dissection of the hippocampus and fornix and a review of the RAT rules. The etymological analysis indicated that the *hippocampus* refers to a sea horse; however, the term also has a mythological background. *Cornu ammonis*, on the other hand, refers to the horns of an Egyptian god. The terminologies present discrepancies regarding the terms derived from *hippocampus* and *cornu ammonis*. Although both terms appear in various terminologies, they are mythonyms that fail to describe the structure they refer to or meet the requirements set by FIPAT.

## Introduction

The hippocampus, located deep in the temporal lobe and surrounded by the cerebral ventricles, plays a fundamental role in emotional regulation, learning, memory, and various cognitive functions (White et al., 2024). The Italian anatomist Julius Caesar Arantius (1530-1589) first described it in the first chapter of his work *De humano foetu liber* (Arantius, 1587), where he proposed the term *hippocampum* due to its similarity to a sea horse. Years later, French anatomists visualized that the hippocampus, macroscopically, presented a different shape to that of a sea horse, which is why it was initially called ram’s horns (Winslow, 1732), and a decade later, it was named cornu ammonis (Garengeot, 1742). Since then, the terms hippocampus and cornu ammonis have become central to medical literature. Initially, both concepts referred to the same structure, but their usage has expanded to include distinct yet related formations (Anand and Dhikav, 2012; Twait et al., 2023; White et al., 2024), a circumstance that can complicate the understanding and function of the structure.

To avoid confusion about the structures that make up the human being, the International Federation of Associations of Anatomists (IFAA) has implemented several programs, including the Federative International Programme for Anatomical Terminology (FIPAT). The latter provides a set of terminologies that are accepted worldwide for the description of anatomical structures. Morphological Sciences use these terminologies as linguistic tools that allow the reader to interpret the content accurately and unambiguously (Duque-Colorado et al., 2023). Thus, the objective of this work was to determine the presence of the terms *hippocampus*, *cornu ammonis* and their variants in *Terminologia Anatomica* (FIPAT, 2019), *Terminologia Neuroanatomica* (FIPAT, 2017) and *Terminologia Histologica* (Federative International Committee on Anatomical Terminology [FICAT], 2008), evaluating their congruence in said terminologies, in addition to analyzing the etymology of both terms and their concordance with the guidelines established by FIPAT.

## Materials and methods

### Review and etymological analysis

We conducted a search in *Terminologia Anatomica* (FIPAT, 2019), *Terminologia Neuroanatomica* (FIPAT, 2017), and *Terminologia Histologica* (Federative International Committee on Anatomical Terminology [FICAT], 2008) for terms containing the concepts *hippocampus*, *cornu ammonis*, and their derivatives. The terms *hippocampus* and *cornu ammonis* underwent review and etymological analysis using a Latin dictionary (Lewis and Short, 1945), the Oxford Latin Dictionary (Oxford Dictionaries, 2012), and the Perseus Digital Library (Crane, 1987). The analysis included a review of the Regular Anatomical Terminology (RAT) rules to assess the consistency of the terms with the guidelines and objectives set by FIPAT.

### Dissection

One cerebral with no macroscopic evidence of disease, belonging to a male adult, was fixed in 10% formalin, after 8 weeks the hemisphere was washed for several hours in fresh water. To preserve the white matter (fornix, anterior column of the fornix and mammillothalamic tract) we used the Klingler method (Castruita et al., 2024). The arachnoid, pia mater and vascular structures were removed, and the hemisphere was placed in a plastic bag and stored in a deep freezer at –20°C for 4 weeks. The hemisphere was thawed at room temperature and washed in running water. Once thawed and during dissections, the samples were preserved in a 70% ethanol solution.

The cortex of the subrostral area, including the paraterminal and paraolfactory gyrus, was carefully removed. Removal of the cingulate gyrus cortex exposed the supracommissural part of the hippocampus and its lateral longitudinal striae on the upper surface of the corpus callosum. The corpus callosum was dissected from the genu to the splenium, along with the septum pellucidum, allowing visualization of the upper surface of the thalamus and the body of fornix.

A No. 10 blade then made cuts along the superior margin of the caudate nucleus superiorly, the isthmus of cingulate gyrus posteriorly, the cerebral peduncle inferiorly, and the posteromedial orbital gyri anteriorly. Then, in a lateral to medial direction, the cortex was removed along with the superficial U-shaped fibers of the insula, identifying the thalamus on the lateral side and the fornix and parahippocampal gyrus on the medial side. Using a No. 15 blade, the white matter of the temporal peduncle was cut approximately 5 mm below the inferior limiting sulcus, identifying the alveus and collateral eminence. The fornix was dissected gradually, starting at the level of the anterior commissure down to the fimbria.

Using a No. 10 blade, we cut the anterior part of the temporal peduncle at the level of the amygdaloid body, allowing identification of the parahippocampal/hippocampal gyrus complex from different angles. In the final step, we dissected the hippocampus from the parahippocampal gyrus along the hippocampal sulcus.

## Results

In *Terminologia Anatomica* (FIPAT, 2019), the term *hippocampus* was detailed with code 5518 as a general concept, with its synonym in US English corresponding to “hippocampal formation,” which encompassed several structures. Similarly, several related concepts were present, including the term *hippocampus proper*, a Latin synonym for the primary term *cornu ammonis* with code 5520, the elements of which are presented in Table 1.

**TABLE 1:** *Hippocampus*, *cornu ammonis* and its derivatives in *Terminologia Anatomica* (FIPAT, 2019).

	Latin term	Latin synonym	UK English	US English	US synonym	Other
5515	Gyrus parahippocampalis		Parahippocampal gyrus	Parahippocampal gyrus		Gyrus hippocampi
5518	*Hippocampus*		*Hippocampus*	*Hippocampus*	Hippocampal formation	
5520	*Cornu ammonis*	*Hippocampus* proper	*Cornu ammonis*	*Cornu ammonis*	*Hippocampus* proper; Ammon’s horn	
5522	Sulcus hippocampalis		Hippocampal sulcus	Hippocampal sulcus		
5523	Alveus hippocampi		Alveus	Alveus		
5524	Fimbria hippocampi		Fimbria of *hippocampus*	Fimbria of *hippocampus*		Fimbria of fornix
5530	Archicortex		Archicortex	Archicortex		Archaecortex; Archecortex; Hippocampal llocortex
5616	Commissura hippocampi	Commissura fornicis	Hippocampal commissure	Hippocampal commissure	Commissure of fornix	Fornix transversus; Psaltarium; Lyre of David
5656	Pes hippocampi		Pes hippocampi	Pes hippocampi		

*Terminologia Neuroanatomica* (FIPAT, 2017) did not include the term *hippocampus*. However, this terminology included more concepts derived from the term in question. Repeated concepts identified with different codes included *formatio hippocampi*, appearing under codes 2176 and 2330. Another term was *hippocampus proprius*, which was detailed as a Latin synonym represented by code 2182, while code 2331 represented a primary term in Latin, whose synonym was *cornu ammonis*. Regarding *cornu ammonis*, in addition to being a Latin synonym for *hippocampus proprius*, it corresponded to several primary terms in Latin *cornu ammonis* 1, *cornu ammonis* 2, *cornu ammonis* 3, and *cornu ammonis* 3h, identified with codes 2339, 2340, 2341, and 2342, respectively. Their Latin synonym, as well as their UK and US English equivalents, used the acronym CA. The details of this paragraph appear in Table 2.

**TABLE 2:** *Hippocampus*, *cornu ammonis* and its derivatives in *Terminologia Neuroanatomica* (FIPAT, 2017).

	Latin term	Latin synonym	UK English	US English	US synonym	Other
425	Rami hippocampales		Branches to hippocampus	Branches to hippocampus		
589	Arteriae hippocampales		Hippocampal arteries	Hippocampal arteries		
590	Arteria hippocampalis posterior		Posterior hippocampal artery	Posterior hippocampal artery		
591	Arteria hippocampalis media		Middle hippocampal artery	Middle hippocampal artery		
592	Arteria hippocampalis anterior		Anterior hippocampal artery	Anterior hippocampal artery		
644	Venae hippocampales longae		Long hippocampal veins	Long hippocampal veins		
645	Vena hippocampalis longa anterior		Anterior long hipocampal vein	Anterior long hipocampal vein		
646	Vena hippocampalis longa posterior		Posterior long hipocampal vein	Posterior long hipocampal vein		
2151	Gyrus parahippocampalis		Parahippocampal gyrus	Parahippocampal gyrus	T5	For subdivision, see Lobus limbicus.
2161	Gyrus parahippocampalis		Parahippocampal gyrus	Parahippocampal gyrus	T5	For subdivision, see Lobus limbicus
2164	Verrucae hippocampi		Hippocampal warts	Hippocampal warts		
2176	*Formatio hippocampi*		*Hippocampal formation*	*Hippocampal formation*	Inner ring of limbic lobe	
2177	Pars precommissuralis hippocampi		Precommissural part of hippocampus	Precommissural part of hippocampus		
2178	Pars supracommissuralis hippocampi		Supracommissural part of hippocampus	Supracommissural part of hippocampus		
2182	Pars retrocommissuralis hippocampi	Hippocampus proprius	Retrocommissural part of hippocampus	Retrocommissural part of hippocampus	Hippocampus proper	
2183	Sulcus hippocampalis		Hippocampal sulcus	Hippocampal sulcus		
2186	Fimbria hippocampi		Fimbria of hippocampus	Fimbria of hippocampus		
2330	*Formatio hippocampi*		*Hippocampal formation*	*Hippocampal formation*		
2331	Hippocampus proprius	Cornu ammonis	Hippocampus proper	Hippocampus proper	Ammon’s horn	
2333	Digitationes hippocampi		Hippocampal digitations	Hippocampal digitations		
2338	*Regiones hippocampi proprii*		*Hippocampal fields*	*Hippocampal fields*		
2339	Cornu ammonis 1	CA1	CA1 field	CA1 field		
2340	Cornu ammonis 2	CA2	CA2 field	CA2 field		
2341	Cornu ammonis 3	CA3	CA3 field	CA3 field		
2342	Cornu ammonis 3h	CA3h	CA3h field	CA3h field		CA4
2343	*Strata hippocampi*	Strata cornus ammonis	*Layers of hippocampus*	*Layers of hippocampus*	Layers of Ammon’s horn	
2351	Interneura hippocamp		Hippocampal interneurons	Hippocampal interneurons		
2356	Substantia alba hippocampi		Fiber connections of hippocampus	Fiber connections of hippocampus		
2358	Neurofibrae muscosa hippocampi proprii		Mossy fibers	Mossy fibers		
2359	Collaterales axonales hippocampi proprii		Axon collaterals of hippocampus proper	Axon collaterals of hippocampus proper	Schaffer collaterals	
2360	Collaterales axonales hilares hippocampi	Via endofolialis	Hilar Schaffer collaterals	Hilar Schaffer collaterals	Endfolial pathway	
2361	Alveus hippocampi		Alveus	Alveus		
2362	Fimbria hippocampi		Fimbria	Fimbria		
2365	Commissura hippocampi	Psalterium	Hippocampal commissure	Hippocampal commissure	Psalterium	Commissura fornicis
2480	Commissura hippocampi	Psalterium	Hippocampal commissure	Hippocampal commissure	Psalterium	Commissura fornicis
2587	Area transitionis amygdalohippocampalis		Amygdalohippocampal transition area	Amygdalohippocampal transition area		
2529	Cortex periamygdaloideus	Area transitionis amygdaloparahippocampalis	Periamygdaloid cortex	Periamygdaloid cortex	Parahippocampal amygdaloid transition area	

In *Terminologia Histologica* (Federative International Committee on Anatomical Terminology [FICAT], 2008), under code H3.11.03.6.01114, the term *hippocampus proprius* or *cornu ammonis* was identified as the primary term in Latin.

According to different texts, as shown in Table 3, the term *hippocampus* from the Greek íππóκαμπoς, refers to a sea horse, which is also a mythological creature. On the other hand, the term *cornu ammonis* was reviewed separately, with *cornu* referring to the protuberances on the head that depicted the gods. Along the same lines, *ammonis* was identified as Ammon, derived from the Greek ’‘Aμμων, the name of a god venerated in Africa, whose cult spread to Greece.

**TABLE 3:** Meaning of the terms *hippocampus*, *cornu*, and *ammonis*.

Dictionary	Term	Meaning
A Latin dictionary (1945)	Hippocampus	-os, i, m., = ıππóκαμπoς, a sea horse: syngnathus hippocampus.
	Cornu	Hard growth on the head of many mammals. The tooth or tusk of an elephant. Horns with the growths that represented the gods.
	Ammon	-onis, [Egypt, Amun] name of Jupiter, worshipped in Africa in the form of a ram (in the present-day oasis of Siwha).
Oxford Latin dictionary (2012)	Hippocampus	-os, [Gk ıππóκαμπoς] a sea horse. A legendary creature associated with the gods.
	Cornu	$\bar{\nu }$s, 1. Animal horn (attributed to certain deities, especially river gods); Hammonis.
	Ammon	-oni, m., also Hammón, Hammon, Hammon. Egyptian god with the form of a ram in Libya; he was identified as Jupiter Hammon.
Perseus Digital Library (2012)	Hippocampus	-os, i, m. = ıππó-καμπoς.
	ıππó-καμπoς	Monster with the body of a horse and the tail of a fish, on which the gods of the sea rode.
	Cornu	Hard, crooked growth on the head.
	Ammon	-onis. Egypt = ’‘Aμμων.
	’‘Aμμων	1. Originally a Libyan or Ethiopian deity; 2. Zeus, adjective of Ammonis; 3. The Greeks called him Zeus Ammon and the Romans Jupiter Ammon.

**FIGURE 1:**
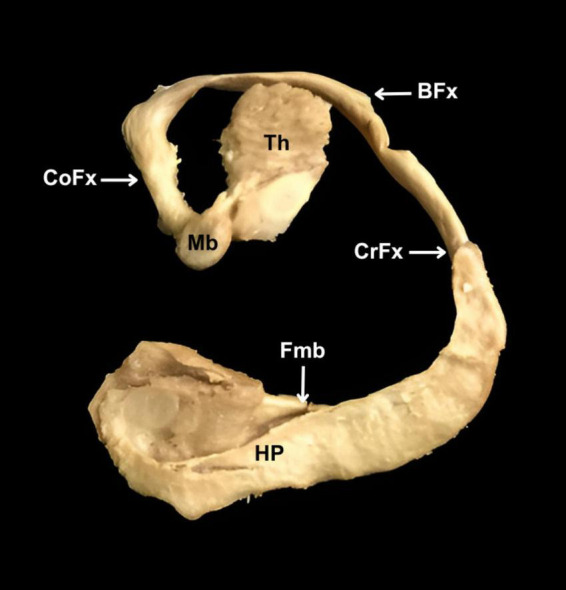
Dissection of hippocampus and fornix. HP, hippocampus. Fmb, fimbria. CrFx, crus of fornix. BFx, body of fornix. CoFx, column of fornix. Mb, mammillary body. Th, thalamus.

The term *hippocampus* etymologically refers to a sea horse. However, during the dissection performed (Figure 1), we observed that the hippocampus does not have this form. For the hippocampus to resemble a sea horse, it must incorporate the fornix. Thus, this term does not comply with numeral four of the RAT rules, which, in turn, causes the words that include or derive from the term *hippocampus* to fail to meet the RAT rules, as outlined in Table 4. Along these lines, the term *cornu ammonis* refers to the horns of an African god called Ammon, making it an eponym with a mythological background. Such a term violates number seven and goes against the purposes of FIPAT.

**TABLE 4:** Regular anatomical terminology (RAT) rules.

1.	That, with a very limited number of exceptions, each structure shall be designated by one term only.
2.	That each term in the official list shall be in Latin, each country shall being at liberty to translate the oficial Latin term into its own vernacular for teaching purposes.
3.	That each term shall be, so far as possible, short and simple.
4.	That the terms shall be primarily memory signs, but shall preferably have some informative or descriptive value.
5.	That structures closely related topographically shall, as far as possible, have similar names.
6.	That differentiating adjectives shall be, in general, arranged as opposites.
7.	That eponyms shall not be used in the Official Nomenclature of Gross or Microscopic anatomy.
8.	That each name must be unique.
9.	That each name shall consist only of nouns and adjectives.
10.	That each name shall have only one noun in nominative case.
11.	That the standard word order shall have nouns following the noun they modify, and adjectives immediately following the noun they modify.
12.	That nouns in genitive case are generally preferable to adjectives when the modifier means “of” an entity rather than “pertaining to” an entity.W3510

## Discussion

“Horum ventriculorum basi, quae intrb ad medium respicit, candida insurgens supereminet, and quasi adnascitur substantia, quz ab inferiori superficie, uelut additamentum extollitur, psalloidique corpori, seu testudini est continua, ac per longitudinem, in anteriora, uersus frontem protenditur inaequalique, ac flexuosa figura praedita est, quae Hippocampi, hoc est marini equuli effigiem rcfert, vel potius, bombycini uernis candidi spinalis medulle initium hinc inde amplexantis” (Arantius, 1587).

The above sentence corresponds to the first description of the hippocampus by Julius Caesar Arantius, considered by Lewis (1923) to be the worst anatomical description in existence. This is because it does not clarify whether the comparison refers to a fish or a beast, and it prevents us from determining with certainty which of its ends represents the head.

Arantius (1587) observation was largely forgotten until two centuries after the publication of the Eustachian tables (Eustachi, 1714) when it regained attention and led to the introduction of the terms ram’s horns (Winslow, 1732) and cornu ammonis horns (Garengeot, 1742). This last term replaced the term ram’s horns without any sound reason, and although it was adopted by several anatomists of the time, Hyrtl (1880) harshly criticized this term and ridiculed it, stating the following: “In order that the organ of the human soul should have no ordinary horns, out of the Cornua arietis were made the Cornua Ammonis, which amount to the same thing.” Later, the hippocampus received more names in different languages, such as gerollte wulst “rolled lump” and kolbe “mass-like structure” (Meyer, 1971). It was even confused with *hippopotamus* by Mayer (1779), and this term was adopted by several German anatomists. Thus, the term hippocampus exemplifies how creativity influences the assignment of names, prioritizing ingenious concepts over precise scientific descriptions (Burdach, 1822).

The term *hippocampus*, with code 5518, is only present in *Terminologia Anatomica* (FIPAT, 2019). Its absence in *Terminologia Histologica* (Federative International Committee on Anatomical Terminology [FICAT], 2008) was to be expected since it is a macroscopic structure. Its absence in *Terminologia Neuroanatomica* (FIPAT, 2017) may be because the US synonym for *hippocampus* in *Terminologia Anatomica* (FIPAT, 2019) is hippocampal formation, a concept listed as the primary term in *Terminologia Neuroanatomica* (FIPAT, 2017) under codes 2176 and 2330. Therefore, *hippocampus* may be equivalent to *formatio hippocampi* (hippocampal formation). However, several studies indicate that the hippocampus is a different structure from the hippocampal formation (Jarrard and Davidson, 1991; Anand and Dhikav, 2012; Schultz and Engelhardt, 2014; Pang et al., 2019).

The hippocampus is a structure comprising the hippocampus proper and the dentate gyrus, both regions coiled around each other (Tatu and Vuillier, 2014). The hippocampus proper is structured mainly from pyramidal neurons, and the dentate gyrus from granule neurons. The hippocampus proper is structured mainly from pyramidal neurons, and the dentate gyrus from granule cells. This region is a site of neurogenesis, particularly in the dentate gyrus, so this structure contributes mainly to its role in learning, memory (Fabiano et al., 2025), time perception (Tatu and Vuillier, 2014), and emotional memory (Maggio and Segal, 2012). On the other hand, the hippocampal formation includes several interconnected regions, namely the hippocampus (hippocampus proper and dentate gyrus), subiculum, and entorhinal cortex (Pang et al., 2019). In this sense, the hippocampal formation covers a broader range of functions related to memory and spatial navigation since the subiculum allows processing and amplifying hippocampal information while the entorhinal cortex integrates and channels cortical information, thus creating a bidirectional flow for memory consolidation and recovery (Böhm et al., 2017; Roy et al., 2017). Consequently, the entorhinal cortex is a key information input and output center (Fabiano et al., 2025).

Considering the above mentioned, there are deficiencies in the terminologies, thinking that if hippocampus and hippocampal formation refer to the same thing, there would be no agreement between Terminologia Anatomica (FIPAT, 2019) and Terminologia Neuroanatomica (FIPAT, 2017), since the same structure has two different names, an aspect that goes against the first numeral of the RAT rules, so a single concept should be considered as the primary term in Latin, being hippocampus or formatio hippocampi. If these are distinct structures, the US synonym must be discarded, as they are referred to by the same name, which contradicts section eight of the RAT rules.

In this same context, another element that generates discrepancies between terminologies is that in *Terminologia Anatomica* (FIPAT, 2019), under code 5520, the primary term is found in Latin *cornu ammonis*, with a synonym in Latin *hippocampus proper*. A completely different condition appears in *Terminologia Neuroanatomica* (FIPAT, 2017), where under code 2331, it is designated as the primary term in Latin, *hippocampus proprius*, with the Latin synonym *cornu ammonis*. This aspect explains the lack of synergy between terminologies, as the same structure is referred to by different names in documents that belong to the same program, such as FIPAT, which aims to serve as a reference for the identification and standardized naming of human structures in the medical and morphological fields. Likewise, for each terminology, the term used in UK and US English is derived from the primary Latin term, resulting in increased confusion in the morphological and educational fields, as scientific literature is predominantly written and published in English.

In *Terminologia Neuroanatomica* (FIPAT, 2017), the regions of the *hippocampus proper* are given by several subfields, which are detailed as main terms in Latin by *cornu ammonis* 1, *cornu ammonis* 2, *cornu ammonis* 3 and *cornu ammonis* 3h. Despite this, their synonyms in Latin, translated into UK and US English, are CA1, CA2, CA3, and CA3h, respectively, acronyms that do not provide references about the structure they represent lack informative value (Duque-Colorado et al., 2024), which does not promote the purposes of FIPAT and goes against the RAT rules.

**FIGURE 2:**
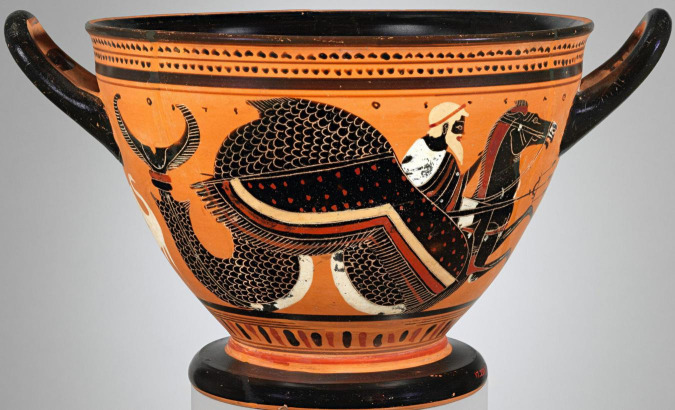
Attic vase painted by the Leagros group in the 6th century BCE, depicting Poseidon riding a hippocampus.

**FIGURE 3:**
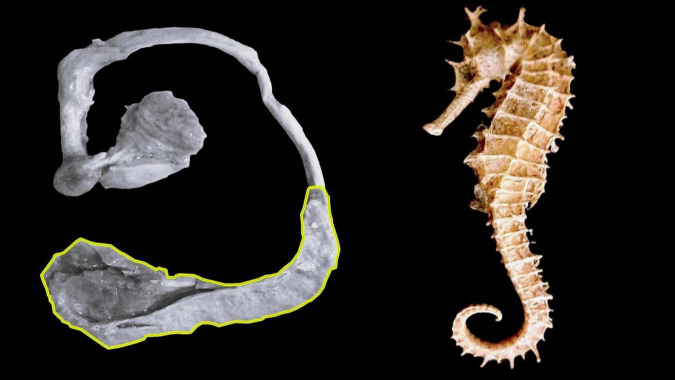
Comparison between hippocampus and seahorse. **(A)** Hippocampus highlighted in yellow. **(B)** Seahorse.

Arantius (1587) proposed the term *hippocampum* for this brain structure because of its similarity to a sea horse. Our results indicated that the term *hippocampus*, which comes from the Greek íππóκαμπoς, arises from the union of íππoς (horse) and καμπoς (sea monster), so although it is associated with a sea horse, it has also been associated with a mythical monster with a fishtail and a horse’s body, on which the sea god Poseidon rode (Figure 2). Thus, the term *hippocampus* corresponds to a zoonym with a mythological background. Despite the above, we agree with some authors (Lewis, 1923; Winslow, 1732; Garengeot, 1742) that the hippocampus at a macroscopic level does not share the same shape as a seahorse, since for them to compare, in addition to considering the hippocampus, the structures that make up the fornix should also be considered (Figure 3).

Although initially, Garengeot (1742) used the term *cornu ammonis* to refer to the hippocampus, it is currently considered a structure that is part of the *hippocampus*, according to *Terminologia Anatomica* (FIPAT, 2019) or of the *formation hippocampi* according to *Terminologia Neuroanatomica* (FIPAT, 2017). According to the description made by Garengeot (1742), the hippocampus had the shape of a ram’s horns, which is why he called it *cornu ammonis* (Ammons’ horns). The etymology of the words tells us that *cornu* refers to protuberances that protrude from the head and are associated with deities (Lewis and Short, 1945). This is because, over time, art and myths have preserved animal parts, such as wings, hooves, and horns, for the gods, with artists using horns as a symbol of power. In this sense, the term *cornu ammonis*, in addition to representing a structure of the hippocampus, refers to the horns of the god Ammon (’‘Aμμων), a supreme deity in Libya and Egypt. Initially, this god appeared with antlers on his head. His depiction changed to ram’s horns when his cult spread to Greece in the 5th century BCE, where he became known as Zeus-Ammon. This suggests that this term is based on an eponym in a mythological context, making it a mythonym.

Currently, the term *cornu ammonis* refers to the regions of the hippocampus, which are visible microscopically but not macroscopically. These fields are classified based on the characteristics of the neurons that compose each region; therefore, *Terminologia Anatomica* (FIPAT, 2019) should not list them.

*Hippocampus* and *cornu ammonis* are not the only terms used that barely make sense. In *Terminologia Anatomica* (FIPAT, 2019), criticisms have been made of different terms, such as *diastema* (Panes and del Sol, 2020), *humor aquosus* (García-Orozco et al., 2023), *fossa* and *fovea* (Alarcón-Apablaza et al., 2024), *calva* (García-Orozco et al., 2024), among other terms. For its part, we consider that *Terminologia Neuroanatomica* (FIPAT, 2017) has received less attention. However, terms included in this document, such as *splenium* (Pearce, 2008), the structures that make up the brainstem (Watson et al., 2019), fornix (Dogan et al., 2022), *thalamus* (García-Cabezas et al., 2021), *corpus amygdaloideum* (Maldonado-Rengel et al., 2021), *substantia chromatophilca* (Duque-Colorado et al., 2023) and *neuron parvum fluorescens* (Valenzuela-Aedo et al., 2024) have also been subject to criticism. Morphological Sciences, Medical Sciences, and Neuroscience are constantly evolving; therefore, it is natural that FIPAT terminologies undergo continuous transformation. Thus, we believe it is appropriate to gradually replace terms unrelated to the structures to which they refer and that the terms included be governed by the 12 principles established in the RAT rules, thus allowing for the construction of contemporary terminologies.

The RAT rules consist of a series of recommendations whose application ensures the use of precise terms for new and known structures. This approach aligns with the objective of the FIPAT terminologies, ensuring accurate and well-defined concepts that facilitate communication among health professionals and enhance teaching-learning processes in morphological areas (Duque-Colorado et al., 2024). Therefore, although various terms have spread and consolidated in the medical, morphological, and teaching fields, we consider it pertinent to re-evaluate the terms mentioned in this chapter.

The first descriptions of human body structures by anatomists were fundamental to the development of modern medicine. Through detailed studies and precise observations, these pioneers not only described previously unknown structures but also laid the foundations for Terminologia Anatomica (FIPAT, 2019), Terminologia Neuroanatomica (FIPAT, 2017), and Terminologia Histologica (Federative International Committee on Anatomical Terminology [FICAT], 2008). While some of these terms have been subject to criticism and revision over time, driven by advances in scientific and cultural understanding, their value and original contribution remain significant. The work of these anatomists not only bequeathed a fundamental structure of knowledge but also highlighted the importance of rigorous observation and systematization of the human body—principles that remain key pillars of medical research today.

## Conclusion

There is no congruence between terminologies to designate the terms hippocampus and cornu ammonis. Both terms are mythonyms, although they originate from different myths, *hippocampus* relates to a sea monster, while *cornu ammonis* refers to an Egyptian god. Although the hippocampus was initially named as such because of its similarity to a seahorse, dissection has shown that it does not have the shape of this marine animal. Consequently, both terms contradict the purposes and objectives of FIPAT. Therefore, advancing further studies in this area would be relevant to implement the necessary modifications in these documents, which regulate uniform communication among health professionals, morphologists, neuroscience professionals, and students.

## Data Availability

The original contributions presented in the study are included in the article/supplementary material, further inquiries can be directed to the corresponding authors.
